# Pathway-dependent brain stimulation responses indicate motion processing integrity after stroke

**DOI:** 10.1093/brain/awaf043

**Published:** 2025-02-04

**Authors:** Michele Bevilacqua, Fabienne Windel, Elena Beanato, Pauline Menoud, Sarah Zandvliet, Nicola Ramdass, Lisa Fleury, Julie Hervé, Krystel R Huxlin, Friedhelm C Hummel, Estelle Raffin

**Affiliations:** Defitech Chair of Clinical Neuroengineering, Neuro-X Institute (INX), École Polytechnique Fédérale de Lausanne (EPFL), Geneva 1202, Switzerland; Defitech Chair of Clinical Neuroengineering, INX, EPFL Valais, Clinique Romande de Réadaptation, Sion 1951, Switzerland; Defitech Chair of Clinical Neuroengineering, Neuro-X Institute (INX), École Polytechnique Fédérale de Lausanne (EPFL), Geneva 1202, Switzerland; Defitech Chair of Clinical Neuroengineering, INX, EPFL Valais, Clinique Romande de Réadaptation, Sion 1951, Switzerland; Defitech Chair of Clinical Neuroengineering, Neuro-X Institute (INX), École Polytechnique Fédérale de Lausanne (EPFL), Geneva 1202, Switzerland; Defitech Chair of Clinical Neuroengineering, INX, EPFL Valais, Clinique Romande de Réadaptation, Sion 1951, Switzerland; Defitech Chair of Clinical Neuroengineering, INX, EPFL Valais, Clinique Romande de Réadaptation, Sion 1951, Switzerland; Defitech Chair of Clinical Neuroengineering, INX, EPFL Valais, Clinique Romande de Réadaptation, Sion 1951, Switzerland; Defitech Chair of Clinical Neuroengineering, Neuro-X Institute (INX), École Polytechnique Fédérale de Lausanne (EPFL), Geneva 1202, Switzerland; Defitech Chair of Clinical Neuroengineering, INX, EPFL Valais, Clinique Romande de Réadaptation, Sion 1951, Switzerland; Defitech Chair of Clinical Neuroengineering, Neuro-X Institute (INX), École Polytechnique Fédérale de Lausanne (EPFL), Geneva 1202, Switzerland; Defitech Chair of Clinical Neuroengineering, INX, EPFL Valais, Clinique Romande de Réadaptation, Sion 1951, Switzerland; Defitech Chair of Clinical Neuroengineering, Neuro-X Institute (INX), École Polytechnique Fédérale de Lausanne (EPFL), Geneva 1202, Switzerland; Defitech Chair of Clinical Neuroengineering, INX, EPFL Valais, Clinique Romande de Réadaptation, Sion 1951, Switzerland; The Flaum Eye Institute and Center for Visual Science, University of Rochester, Rochester, NY 14620, USA; Defitech Chair of Clinical Neuroengineering, Neuro-X Institute (INX), École Polytechnique Fédérale de Lausanne (EPFL), Geneva 1202, Switzerland; Defitech Chair of Clinical Neuroengineering, INX, EPFL Valais, Clinique Romande de Réadaptation, Sion 1951, Switzerland; Clinical Neuroscience, University of Geneva Medical School, Geneva 1206, Switzerland; Defitech Chair of Clinical Neuroengineering, Neuro-X Institute (INX), École Polytechnique Fédérale de Lausanne (EPFL), Geneva 1202, Switzerland; Defitech Chair of Clinical Neuroengineering, INX, EPFL Valais, Clinique Romande de Réadaptation, Sion 1951, Switzerland

**Keywords:** hemianopia, direction discrimination, transcranial magnetic stimulation-electroencephalography (TMS-EEG), cortico-cortical paired associative stimulation (ccPAS), Granger Causality

## Abstract

Homonymous hemianopia (HH), a common visual impairment resulting from occipital lobe lesions, affects a significant number of stroke survivors. Intensive perceptual training can foster recovery, possibly by enhancing surviving visual pathways. This study employed cortico-cortical paired associative stimulation (ccPAS) to induce associative plasticity within the residual and bidirectional primary visual cortex (V1)-middle temporal area (MT) pathways in stroke patients. We used ccPAS, which is thought to tap into Hebbian-like spike-timing dependent plasticity, over a motion processing pathway in stroke patients to transiently improve visual motion discrimination in their blind field.

Sixteen stroke patients participated in this double-blind, crossover study comparing the effects of bidirectional ccPAS (V1-to-MT or MT-to-V1) on motion discrimination and EEG-Granger Causality. Additionally, we explored potential multimodal sources of inter-individual variability.

Results showed that MT-to-V1 ccPAS enhanced motion direction discrimination, but the expected electrophysiological increase in top-down MT-to-V1 inputs was observed only in patients who showed improvement in motion discrimination. Good responders to MT-V1 ccPAS also demonstrated improved functional coupling between the cortical motion pathway and other relevant areas in the visual network, as well as more preserved ipsilesional V1-MT structural integrity.

These findings indicate that targeted ccPAS can effectively engage functionally relevant residual visual pathways in stroke-affected brains, potentially offering new avenues for patient stratification and visual recovery strategies.

## Teaser

Bevilacqua *et al.* explore whether a novel brain stimulation method—cortico-cortical paired associative stimulation (ccPAS)—can improve vision in stroke patients with occipital lobe damage. They show that ccPAS can enhance motion direction discrimination and strengthen connections in visual pathways, highlighting its potential to aid visual recovery.

## Introduction

Stroke is the second-leading cause of death and the third-leading cause of death and disability combined (as expressed by disability-adjusted life-years lost—DALYs) in the world.^1^ Among the survivors, 30% will suffer from a visual deficit.^2-4^ The most common form, homonymous hemianopia (HH), implies loss of vision on the same side of the visual field of both eyes, secondary to occipital lobe lesions (45% of the patients). Spontaneous recovery from HH is possible in the first 3 to 6 months, but only 15% of stroke survivors will fully recover their initial visual field.^5,6^

Although most of the clinical options are compensatory, some degrees of recovery can be obtained using intensive perceptual training.^7-11^ This restorative approach is thought to rely on: (i) the function of surviving circuitry in the primary visual cortex (V1), with receptive fields that are located close to the visual field border; or/and (ii) alternative pathways left intact after the stroke, connecting the lateral geniculate nucleus (LGN) to the extra-striate medio-temporal (MT) area for instance.^12-15^ These alternative pathways explain why despite the absence of conscious visual perception, humans and monkeys with V1 lesions remain able to respond to moving or flickering visual stimuli within the scotomas.^16-22^ Many neurons of the LGN that belong to the lesion projection zone, degenerate in the first months after V1 lesions.^23,24^ However, LGN projections to MT often appear to be preserved.^12,25^ Robust responses have been recorded from all layers of the LGN after V1 lesion,^26^ suggesting that this archaic direct pathway might be involved in residual vision and potentially in recovery. Some authors have hypothesized that residual vision could be improved through training^27^ and that unconscious vision might translate into conscious vision.^28^

In this study, we rather posit that the residual visual function enabled by spared, perilesional V1 neurons might enable plastic changes supporting partial recovery of vision through cortico-cortical pathways. Horizontal excitatory connections in V1 extends several millimetres across the cortex and link cells with non-overlapping receptive fields.^29,30^ These cells have been shown to increase in density shortly after V1 damage, both within and across the lesion projection zone. This causes the receptive fields of cells inside the lesion projection zone to shift outwards, toward the border of the scotoma.^31^ As a result, although being fewer in number, the residual connections between V1 and MT might be more excitable and represent an interesting substrate for visual field recovery. In line with this, Hagan *et al*.^32^ found changes in the strength of connectivity units in MT, probably contributing to reinforcing the weight of the residual from MT to residual neurons in V1. Additionally, long-term potentiation appears to be strengthened near the lesion border after V1 lesions,^33,34^ suggesting that cortico-cortical connections are prone to reorganize.^35,36^ In contrast, the capacity to develop new thalamo-cortical connections that are able to bypass the lesion is very limited in adults.^35,37^

For all these reasons, we investigated the possibility of enhancing plasticity in the V1-MT pathway to transiently modulate direction discrimination performance in the blind field of stroke patients. To do so, we employed cortico-cortical paired associative stimulation (ccPAS) to promote associative plasticity between the residual V1 neurons and MT, which is based on the Hebbian principle of spike-timing dependent plasticity (STDP). The ccPAS technique involves the repeated administration of transcranial magnetic stimulation (TMS) pulse pairs to two interconnected cerebral regions, with an optimal interstimulus interval (ISI) between pulses.^38^ This protocol aims to synchronize the activation of presynaptic neurons in one site with the stimulation of postsynaptic neurons in the other, enhancing or diminishing the neural pathway’s strength between these areas.^39^ The associative coupling of pre- and postsynaptic activity is crucial for STDP, contributing to the modification of functional and effective connectivity within the targeted networks.^40,41^ Applied to the healthy cortical motion processing pathway, results showed that when participants received MT-V1 ccPAS (a first TMS pulse over MT, the second over V1), motion perception improved,^42^ along with an increase in top-down MT to V1 connectivity.^43^ In the present study, we investigated whether the same applies to patients with lesions affecting V1. We measured motion direction discrimination of stimuli individually located in the blind field of patients and recorded EEG activity during the motion direction discrimination task and in response to TMS over residual V1 and MT before and after ccPAS. Because of the crucial role of feedback re-entrant MT-to-V1 projections and based on our previous results in healthy participants, we anticipated beneficial effects of MT-V1 ccPAS on motion discrimination performances compared with the reverse ccPAS condition (V1-MT ccPAS) in occipital stroke patients.

With the aim of identifying individual predictors of behavioural effects, we investigated the changes in connectivity strength from one area to the other one as well as the amount of residual structural fibres connecting V1 and MT. We predicted that individuals with more surviving connecting fibres and with a dynamic functional coupling between the two areas would benefit more from the intervention. Given that visual retraining paradigms in patients often rely on motion processing^10,44,45^ we believe that the individual response to MT-V1 ccPAS, indexing the capacity for pathway-specific cortical reorganization in each individual patient, could be used to predict the individual response to visual rehabilitation.

## Materials and methods

### General procedures

**Figure 1:**
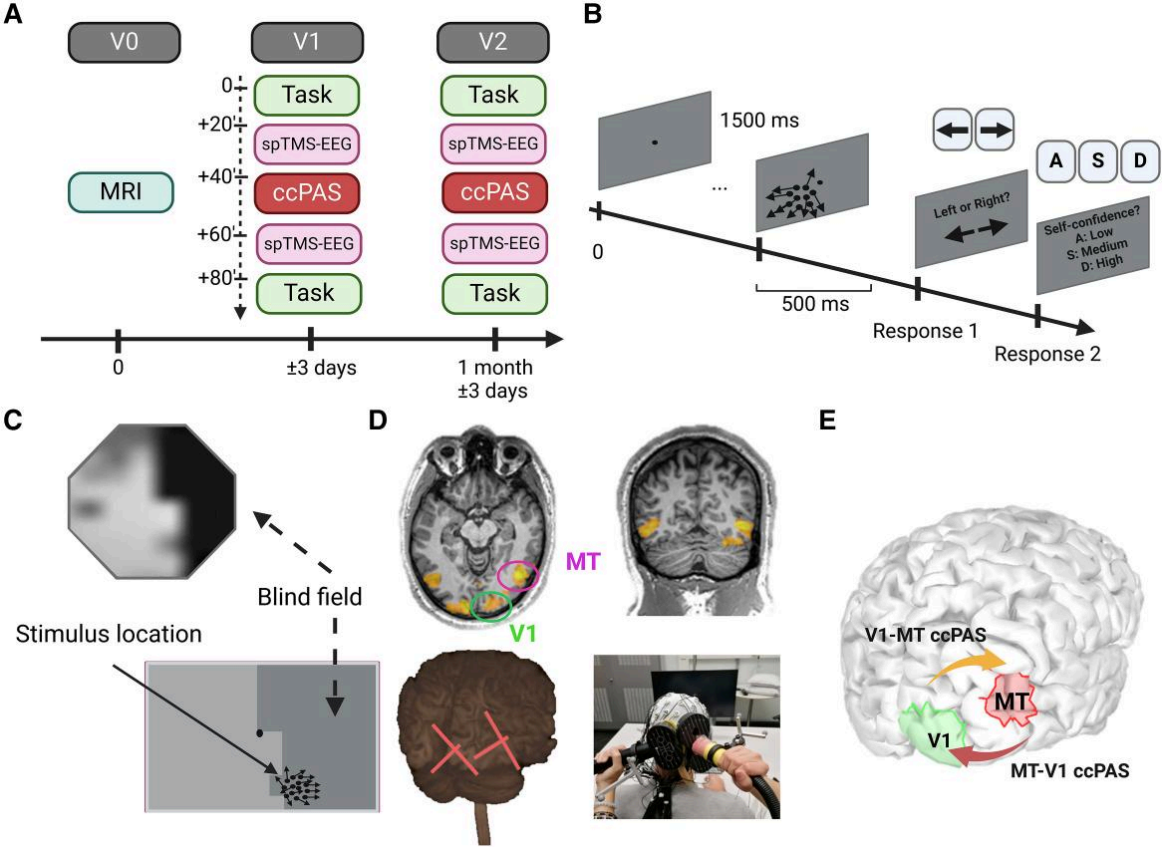
**Protocol details**. (**A**) Timeline of the experiment. (**B**) The motion direction discrimination and integration task used before and after the ccPAS interventions. (**C**) Example of stimulus location based on each individual’s visual field maps. (**D**) Activation maps obtained from the individual MT functional localizer, online neuro-navigation and coil positioning. (**E**) V1-MT ccPAS (yellow arrow) and MT-V1 ccPAS (red arrow). ccPAS = cortico-cortical Paired Associative Stimulation; MT = middle temporal area; spTMS = single-pulse transcranial magnetic stimulation; V1 = primary visual cortex.

This double-blinded and crossover study involved two sessions of 3 h each with a 1 month interval, only differing by the type of ccPAS intervention applied to the patients (randomly MT-V1 ccPAS or V1-MT ccPAS). Prior to the first experimental session, patients underwent an MRI session encompassing functional and anatomical scans and were familiarized to the motion direction discrimination task. Seven patients were tested at Biotech Campus (Geneva, Switzerland) and another subgroup of nine patients were tested at the Clinique Romande de Réadaptation (Sion, Switzerland). Task performances, task-EEG (see Supplementary material) and EEG responses to single pulse TMS over ipsilesional V1 and MT were measured at baseline and after ccPAS (Fig. 1A). At the end of both experimental sessions, patients were asked to fill in a short questionnaire related to sensations and unintended effects of TMS.

After the ccPAS experiment, these patients were enrolled in a visual training protocol involving other brain stimulation modalities. These data will be reported in a separate manuscript.

### Patients

Sixteen adult patients were enrolled at least 1 week after stroke-induced occipital damage (verified using structural MRIs), with reliable 24-2 Humphrey Visual Field (HVF) perimetry. Mean time since stroke was 12.5 months (range: 1–60 months). Exclusion criteria were unreliable HVFs, neglect, neurologic disease unrelated to occipital stroke, use of neuroactive drugs, and any contraindication to MRI or to TMS.^46^ The mean (± SD) age was 59.75 ± 11.55 years, range 34–78; 18.75% were female, 81.25% male. Eight patients had left-sided homonymous visual field loss and eight right-sided homonymous visual field loss (see Table 1 for patients’ characteristics and demographics and see Supplementary Fig. 4 for heat map of lesion frequency).

**Table 1:** Patients’ characteristics and demographics

ID	Age (years)	Sex	Lesion	Lesion side	Time since stroke (months)	MMSE
P101	34	F	Cortical	Left	12	27
P102	62	M	Cortical	Right	12	28
P103	74	M	Cortical	Left	60	25
P104	62	M	Cortical	Right	12	27
P105	66	M	Cortical + subcortical	Right	30	27
P106	68	M	Cortical	Right	8	29
P107	53	M	Cortical	Left	2	30
P201	69	M	Cortical + subcortical	Left	28	25
P202	40	F	Cortical	Left	11	28
P203	59	M	Cortical	Right	4	28
P204	63	M	Cortical	Right	4	29
P205	51	M	Cortical	Left	3	30
P206	56	M	Cortical	Right	11	30
A201	55	M	Cortical	Right	1	29
A202	78	F	Cortical	Left	1	21
A203	66	M	Cortical	Left	1.5	29

F = female; M = male; MMSE = Mini-Mental State Examination.

All patients provided informed, written consent prior to the experiment. This study is part of a registered trial (ClinicalTrials.gov: NCT05220449), which was approved by the local Swiss Ethics Committee (2017-01761) and performed in accordance with the Declaration of Helsinki.

### Behavioural task

We used a well-established two-alternative, forced-choice, global direction discrimination and integration task (150 trials in total), as previously described.^10,47-50^ Subjects were asked to discriminate the left–right direction of the motion of random-dot stimuli (Fig. 1B). They performed 150 trials, with each trial initiated by fixation of a small spot of light within an electronic window 2 × 2° in size. Steady fixation of this target for 1000 ms resulted in a tone signalling the onset of stimulus presentation during which subjects were required to maintain fixation. A break in fixation during stimulus presentation produced a loud, 1 s tone and termination of the trial. After 500 ms, the stimulus and fixation spot disappeared, and subjects were required to indicate whether they perceived the global direction of motion of the stimulus to be toward the right or the left by pressing the right or left arrow key on a computer keyboard placed in front of them. Correct and incorrect responses were signalled by different computer-generated tones, so that the subjects instantly knew whether they performed correctly or not. The sequence of presentation of rightward and leftward drifting stimuli was randomized. The degree of difficulty was increased with task performance by increasing the range of dot directions within the stimulus using a 3:1 staircase design. For every three consecutive correct trials, direction range increased by 40°, while for every incorrect response, it decreased by 40°.

Stimuli consisted of a group of black dots with a density of 2.6 dots/(°)^2^ in a 5° diameter circular aperture, moving at a speed of 10°/s for a lifetime of 250 ms (half the stimulus duration). Self-confidence was rated (low/medium/high) after each trial. The stimuli were individually placed close to the border of the scotoma (see Fig. 1C) while keeping performances around 70% accuracy (mean ± SD = 69.43 ± 5.6% correct). We ensured stable performance by comparing their behavioural score measured on the day of the experiment to the score measured 1 day before.

The task was implemented in MATLAB 2019b (version 1.8, The MathWorks, Inc., USA) coupled with an EyeLink 1000 Plus Eye Tracking System (SR Research Ltd.) sampling at a frequency of 1000 Hz to control gaze and pupil movements in real time. The task was projected onto a mid-grey background liquid crystal display (LCD) projector (1024 × 768 Hz, 144 Hz). During the whole experiment, the participants sat on a chair, with the head leaning on the chinrest, in front of the computer screen, centred 47 cm from the eyes.

### Behavioural analyses

We computed direction range thresholds by fitting a Weibull function to the percentage of correct performance at each stimulus level and finding the stimulus value (i.e. direction range), resulting in 75% correct performance. Direction range (DR) thresholds were then normalized by the maximum range (360) to produce normalized direction range (NDR) thresholds (e.g. Saionz *et al*.^49^) using the following formula:


(1) $$NDR\;\;threshold(\text{\%})=\left[\frac{\left(360^{\circ }-Weibull\;fitted\;DR\right)}{360^{\circ }}\right]\times 100$$

### Single pulse transcranial magnetic stimulation

Biphasic single pulses TMS over V1 and MT were sent with an antero-posterior followed by postero-anterior current in the brain (AP-PA) through a MC-B65-HO butterfly coil (MagVenture A/S) plugged into a MagPro XP TMS stimulator (MagVenture A/S). Pulse duration was 300 μs, delivered through continuous monitoring using the Localite neuronavigation software (Localite GmbH, Germany).

To determine stimulation sites, we used an individual functional MT localizer (see Supplementary material) performed prior to the TMS-EEG exam, combined with the location of the phosphene hotspot for V1 and MT if possible (4/16 for V1 and 2/16 for MT). The functional localizer resulted in bilateral clusters over V1 and MT in all patients (see Fig. 1D). To determine stimulation intensities, we evaluated the phosphene threshold^51^ on both V1 and MT. If the participants reported phosphenes we set the stimulation intensity at 90% of the phosphene threshold. If no phosphenes could be evoked, we used 65% of the maximal stimulator output (MSO) for V1 and 60% for MT in the remaining participants to maximize signal-to-noise ratio and comfort during the exam. This resulted in a mean stimulation intensity for V1 of 67 ± 8% MSO, and for MT, 59 ± 6% MSO. On each area, 90 TMS pulses were performed with an inter-pulse interval of 4 ± 1 s. Stimulation intensities were re-evaluated at the beginning of the second session and adjusted if needed.

### ccPAS interventions

ccPAS was delivered via two independent TMS stimulators externally triggered with Signal (Digitimer, Cambridge Electronic Design). V1 was stimulated using the same stimulator/coil combination of the single pulse TMS-EEG recordings, i.e. with a MagVenture MagPro XP stimulator (MagVenture A/S) connected to an MC-B65-HO coil. The ipsilesional MT area was stimulated with a MagVenture MagPro X100 stimulator (MagVenture A/S), pulse duration 400 μs, biphasic, connected to a smaller coil to allow precise anatomical targeting, i.e. the MC-B35 coil. In Sion, the TMS stimulator used to target MT during ccPAS was a Magstim Rapid^2^ (The Magstim Company Ltd.), pulse duration 400 μs, biphasic. The rest of the set up was exactly the same between the two sites.

The same coil positions used for single TMS were applied for the ccPAS procedure, with both coils being neuronavigated (Fig. 1D, bottom left panel). The same intensities were used for MT. For V1, due to the usage of a different coil, the stimulation intensity was recalibrated to match the single pulse TMS condition; 90 pairs of biphasic stimuli were continuously delivered at a rate of 0.1 Hz for 15 min. The coil handle for MT was held tangentially to the scalp and pointed downwards at an angle of 120° ± 5 clockwise.

We compared two types of ccPAS, differing by the order of the two pulses to explore direction-specific effects of ccPAS in the motion processing network. In the MT-V1 ccPAS condition, the first TMS pulse was applied to MT followed by a pulse to V1. In the forward V1-MT ccPAS condition, the TMS pulses order was reversed; the first pulse was administered to V1 and the second to MT (Fig. 1E). The ISI was set to 20 ms for both ccPAS conditions, because it corresponds to the time delays of MT-V1 back projections.^52,53^ This timing is critical to create sequential presynaptic and postsynaptic activity in the network, and to generate the occurrence of STDP.^40,54^

### EEG recordings

EEG was recorded during single pulse TMS stimulation using a 64-channel, TMS-compatible system (BrainAmp DC amplifiers and BrainCap EEG cap, Brain Products GmbH) with the ground electrode at Fpz, the reference electrode at Cz and the Iz electrode added to the international standard 10–20 layout. Electrode impedances were adjusted and kept under 5 kΩ using conduction gel. The impedance levels were checked throughout the experiment and corrected if needed during breaks between the recordings. The signal was recorded using direct current (DC) mode, filtered with a 500 Hz anti-aliasing low-pass filter, and digitalized at 5 kHz sampling frequency. We used active noise cancellation intra-auricular earphones (Bose QC 20) to mask the TMS click which is susceptible to evoke auditory responses on the ongoing EEG activity. The sound level was adjusted for each subject, so that the TMS click delivered became barely audible without any discomfort for the participant. A thin layer of soft plastic was placed on the coil surface to dampen both sensory and auditory feedback to the patients.

### TMS-EEG pre-processing

EEG analyses were performed on the EEG recordings of the 90 single pulse sessions over V1 and MT. Pre-processing was computed in MATLAB, using the EEGLAB toolbox, the open source TMS-EEG Signal Analyser (TESA) plugin^55^ and the Brainstorm plugin^56^ (see Supplementary material for the task-EEG pre-processing and analyses).

Detection of bad channels was performed using the EEGLAB built-in function. Then, the raw EEG signal was epoched in a window of (−0.5, 1) s around the stimulation pulse onset, and demeaned. Afterwards, a window of (−5, 25) ms around the pulse onset was removed, to remove the TMS artefact, and the missing data was interpolated using a cubic function by considering the data 5 ms before and after the removed TMS artefact window. EEG data were then down-sampled from 5000 to 1000 Hz, and bad epochs (huge rubbing artefacts or undefined, significant noise) were removed by visual inspection. On average, 8 of 90 epochs were removed per recording. Interpolated data in the TMS artefact window were removed again, in order to compute the first round of independent component analysis (ICA), aiming to eliminate components of the pulse artefact. This first ICA was computed by first performing a principal component analysis (PCA) compression using the TESA built-in function and then performing a symmetric fast-ICA with hyperbolic tangent as the contrast function. The artefact components were removed manually by visual inspection. On average, 9 (±5) of 64 components were removed. The EEG signal was re-interpolated in the removed window as described previously and frequency filtered with a bandpass filter between 1 and 80 Hz, with a 4th degree Butterworth notch filter between 48 and 52 Hz to remove the power line noise. Data belonging to the artefact window were removed once more as described earlier, and a second round of fast-ICA was computed to remove other types of artefacts, such as eye movement, blinking, acoustic artefacts and small head movement.^55^ On average, 8 (±3) of 64 components were removed. Finally, the EEG signal was spatially filtered using a common average reference (CAR) filter.

### TMS-EEG local/remote source activity

Source reconstruction for each TMS-evoked potential was performed following the default procedure proposed in Brainstorm (version 23-Mar-2022) software^56^ together with the OpenMEEG Boundary Element Method (BEM) plugins. First, each individual was assigned to their personal head model obtained by segmentation of the T1-weighted MRI, including the cortex and head meshes (15 000 and 10 000 vertices, respectively). The forward model was then computed using the symmetric BEM developed in OpenMEEG freeware, using default values for conductivity and layer thickness.^57^ The locations of the electrodes were individually co-registered on the head model using the default 10–20 layout electrode location. For each of the single pulse TMS selected epochs, the source level activation was computed using a minimum norm imaging linear method with sLORETA (standardized low-resolution brain electromagnetic tomography) as the inverse model. The dipole orientation of the source model was defined as unconstrained to the cortex surface. Source orientation was kept orthogonal to the cortical surface and source amplitude was estimated using the default values of the Brainstorm implementation of the whitened and depth-weighted linear L2-minimum norm solution.

To extract local source activity (LSA) power, regions of interest (ROIs) were created on each patient’s anatomy using the individual TMS coordinates for V1 and MT (see earlier, ‘Single pulse TMS’ section), covering about 200 vertices of cortical mesh for V1 and 200 for MT. LSA power was then computed for each cortical target by averaging the absolute, smoothed (using a spatial smoothing filter with full width at half maximum of 5 mm) and normalized (*z*-score against baseline) source activity within its corresponding ROI. Grand average LSA power was finally calculated for each stimulation site and ccPAS condition by averaging LSA power across subjects. The focus of the study was on LSA early components (<200 ms after the onset).

### TMS-EEG connectivity analysis

We explored effective connectivity at different frequency ranges using spectral Granger Causality, which is a metric of directed interareal influence^58^ between our two ROIs (ipsilesional MT and V1).^52^ Details about the Granger Causality computation can be found in the Supplementary material.

### Functional MRI

Whole-brain MRI was done on a 3-Tesla Siemens scanner available at Fondation Campus Biotech Genève (FCBG), Geneva, Switzerland, or on the same scanner at Hopital du Valais, Sion, Switzerland. High-resolution anatomical images were acquired for anatomical references using magnetization prepared rapid acquisition gradient echo (MPRAGE) inversion time = 900 ms, voxel size = 1 × 1 × 1 mm^3^. Task-related activity was measured with one run of approximatively 700 scans using T2*-weighted blood-oxygenation level dependent (BOLD) effect, a gradient echo-planar imaging protocol and the following parameters: echo time (TE) = 30 ms, repetition time (TR) = 1000ms, flip angle = 90, voxel size = 3 × 3 × 2 mm^3^, field of view = 204 mm × 204 mm, matrix size = 68 × 68 and 37 axial slices each of 2 mm thickness.

fMRI data were analysed using the Statistical Parametric Mapping toolbox (SPM12b, Wellcome Trust Center, London, UK, http://www.fil.ion.ucl.ac.uk/spm), implemented in MATLAB 2019b (The Mathworks, Inc.). The complete pre-processing and first-level processing pipelines can be found in the Supplementary material. We conducted a physio-physiological interaction (PhPI) analysis to measure pre-/post-difference in coupling between the ipsilesional V1-MT connection and the rest of the brain.^58-60^ To do this, we determined the seed brain regions on the basis of the individual V1 and MT clusters from the General Linear Model analysis. Significant clusters survived false discovery rate [FDR(cluster)] corrections at *P* < 0.05.

### Structural MRI

Diffusion-weighted imaging (DWI) data were acquired using a pulsed gradient spin echo sequence with the following parameters: TR = 5000 ms; TE = 77 ms; slices = 84; field of view = 234 × 234 mm^2^; voxel resolution = 1.6 × 1.6 × 1.6 mm^3^; slice thickness of 1.6 mm; readout bandwidth = 1630 Hz/pixels; 64-channel head coil; GeneRalized Autocalibrating Partial Parallel Acquisition (GRAPPA) acceleration factor = 3. Seven T2-weighted images without diffusion weighting (b0; b = 0 s/mm^2^) were acquired, including one in the opposite phase encoding direction. A total of 101 images with non-collinear diffusion gradient directions distributed equidistantly over the half-sphere and covering five diffusion-weighting gradient strengths were obtained (b-values = 300, 700, 1000, 2000, 3000 s/mm^2^; shell-samples = 3, 7, 16, 29, 46). Structural diffusion images were pre-processed by means of FMRIB Software Library^61^ and MRtrix^62^ software (see Supplementary material for the description of the pre-processing steps). Streamlines information between V1, MT and the thalamus were extracted from the tractograms, using two approaches. V1 and MT were derived from the functional localizer, using the individual thresholded activation clusters in the ipsi- and contralesional hemisphere. The thalamus was extracted from the Destrieux atlas parcellation, output of the recon-all function of Freesurfer on the T1-weighted image.^63^ All masks were registered to the average b0 image using Advanced Normalization Tools (ANTs). The function tckedit^62^ from MRtrix was finally used to extract specific streamlines passing through either V1 and MT, V1 and the thalamus or MT and the thalamus. The sum of the weights or the average fractional anisotropy (FA) along these tracts were used as indicators of cross-sectional area and integrity, respectively.

### Statistics

NDR thresholds were entered into a mixed ANOVA that included Time (Pre versus Post) and ccPAS type (Forward versus Backward) as within-subject factors and ccPAS order (first Forward versus first Backward) as a between-subject factor. *Post hoc t*-tests (Tukey’s corrected for multiple comparisons) were performed when appropriate and significance was defined for *P*-values <0.05. A median split was applied to the NDR changes to classify patients into good or bad responders to MT-V1 ccPAS. Significant differences in LSA curves as well as in connectivity strength/frequency-resolved Granger Causality were evaluated within-subjects through non-parametric, cluster-based, permutation testing, excluding frequency-wise outliers (data > 90 percentile).^64^ Additionally, we entered the variables that discriminated good from bad responders to ccPAS into a stepwise regression model to explain the inter-individual variability in behavioural changes after MT-V1 ccPAS (*P*-value for entry: 0.05, for removal: 0.1). The variables were the individual MT-to-V1 effective connectivity values derived from Granger Causality, the changes in local perilesional source activity in response to TMS, the beta-weights extracted from the PhPI analyses in the ipsilesional LGN and the FA value in the V1-MT tract derived from DWI.

## Results

A total of 16 stroke patients were recruited in this double-blinded crossover study. Two participants dropped out after the first session, resulting in 16 datasets for V1-MT ccPAS and 14 datasets for MT-V1 ccPAS. The dataset for one subject that dropped out after the first session (A202, session V1-MT ccPAS) was not included in the study due to the bad data quality.

### MT-V1-ccPAS improves motion perception in the blind field

**Figure 2:**
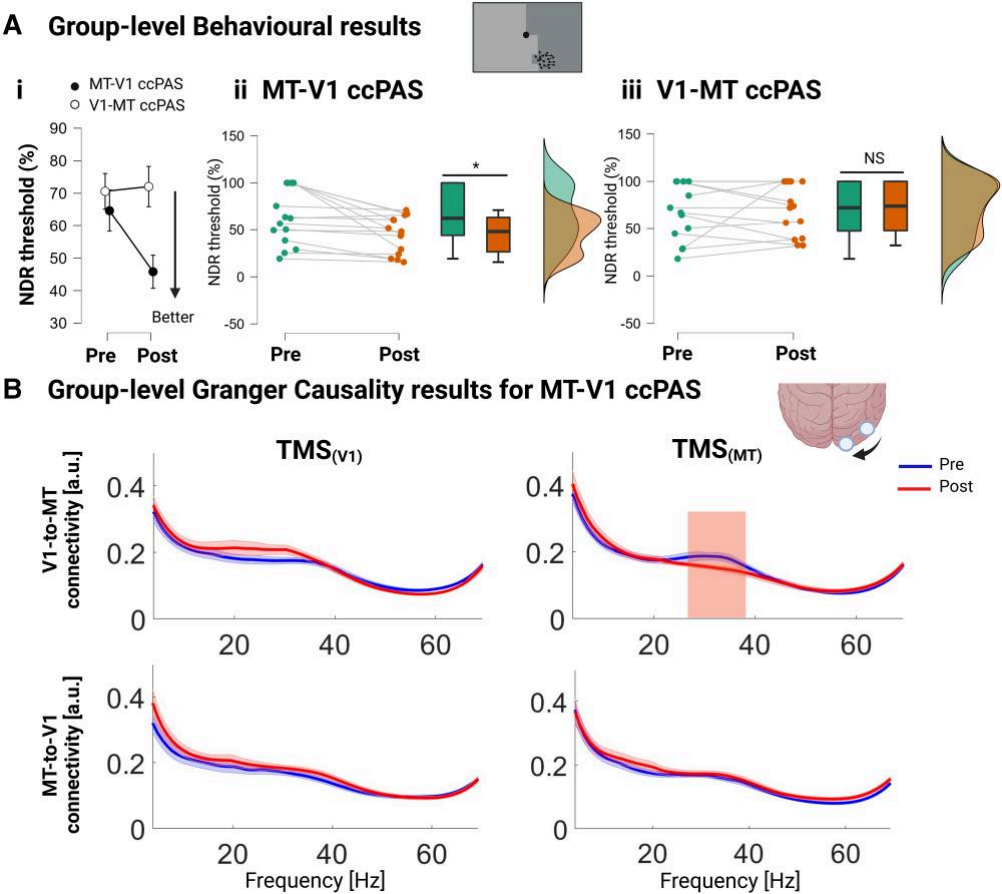
**Group-level results**. [**A**(**i–iii**)] Group-level behavioural changes in normalized direction range (NDR) thresholds. (**i**) NDR values (mean ± SD) for the two ccPAS conditions [repeated measures ANOVA: significant ccPAS type × Time interaction, *F*(1,13) = 6.7, *P* = 0.022]. (**ii**) Individual subjects—changes in NDR thresholds for MT-V1 ccPAS [*post hoc* pre versus post: *t*(14) = 3.3, *P* = 0.006]. (**iii**) Individual subjects—changes in NDR thresholds for V1-MT ccPAS [*post hoc* pre versus post: *t*(15) = −0.24, *P* = 0.81]. (**B**) Group-level Spectral Granger Causality of MT-V1 ccPAS when single pulse transcranial magnetic stimulation (TMS) was given to V1 (*left*) or to MT (*right*). Shaded areas indicate periods of significant differences between pre and post using non-parametric, cluster-based, permutation tests (10 000 permutations, *P* < 0.05), excluding frequency-wise outliers (>90th percentile). ccPAS = cortico-cortical paired associative stimulation; MT = middle temporal area; SD = standard deviation; V1 = primary visual cortex.

The repeated measure ANOVA analysis on the NDR thresholds revealed a significant ccPAS type × Time interaction [*F*(1,13) = 6.7, *P* = 0.022; Fig. 2A(i)]. *Post hoc* comparisons showed that only Backward ccPAS significantly decreased NDR thresholds [Backward: *t*(14) = 3.3, *P* = 0.006, Forward: *t*(15) = −0.24, *P* = 0.81; Fig. 2A(ii and iii)]. There was no main effect of ccPAS [*F*(1,13) = 3.6, *P* = 0.08], Time [*F*(1,13) = 4.01, *P* = 0.07] or ccPAS order [*F*(1,13) = 6.3 × 10^−4^, *P* = 0.98]. Importantly, the baseline level was stable as revealed by the absence of difference between the performances measured the day before and on the first day of the experiment [*t*(15) = −1.54, *P* = 0.14] (Supplementary Fig. 1). Additionally, we looked at changes in reaction times and confidence levels. No significant differences in reaction times and confidence levels were found between the two types of ccPAS intervention (Supplementary Fig. 2).

To understand the neural correlates of MT-V1 ccPAS-induced improvements at the group level, we explored changes in effective connectivity using spectral Granger Causality, which reflects frequency-resolved directed interareal influences, focusing on the bidirectional V1-MT connections after single pulse TMS of V1 and MT (Fig. 2D, group-level Granger Causality results for V1-MT ccPAS can be found in Supplementary Fig. 3).

Results showed a significant decrease in re-entrant V1-MT effective connectivity in the High Beta frequency range (27–36 Hz) after MT single pulse stimulation. No significant changes were observed in connectivity strength with the other stimulated area/direction combinations. None of the time-frequency and effective connectivity comparisons were significant on the task-EEG data (see Supplementary material, ‘Results’ section).

### Differences between good responders and bad responders to MT-V1 ccPAS

Given the high inter-individual variability in the behavioural responses to MT-V1 ccPAS and our strong hypothesis on the involvement of the targeted re-entrant MT-to-V1 inputs, we conducted subgroup analyses based on a median split of the changes in NDR thresholds after MT-V1 ccPAS. This resulted in seven ‘good responders’ and eight ‘bad responders’. We compared the changes in Granger Causality of MT-to-V1 and local EEG source activity in response to single pulse TMS of the perilesional V1. Additionally, we compared to what extent the V1-MT pathway was coupled to other brain regions using whole-brain fMRI PhPI analyses applied to the BOLD data recorded during motion direction discrimination. We also compared the V1-MT pathway’s structural integrity based on DWI at baseline in these two subgroups of patients.

**Figure 3:**
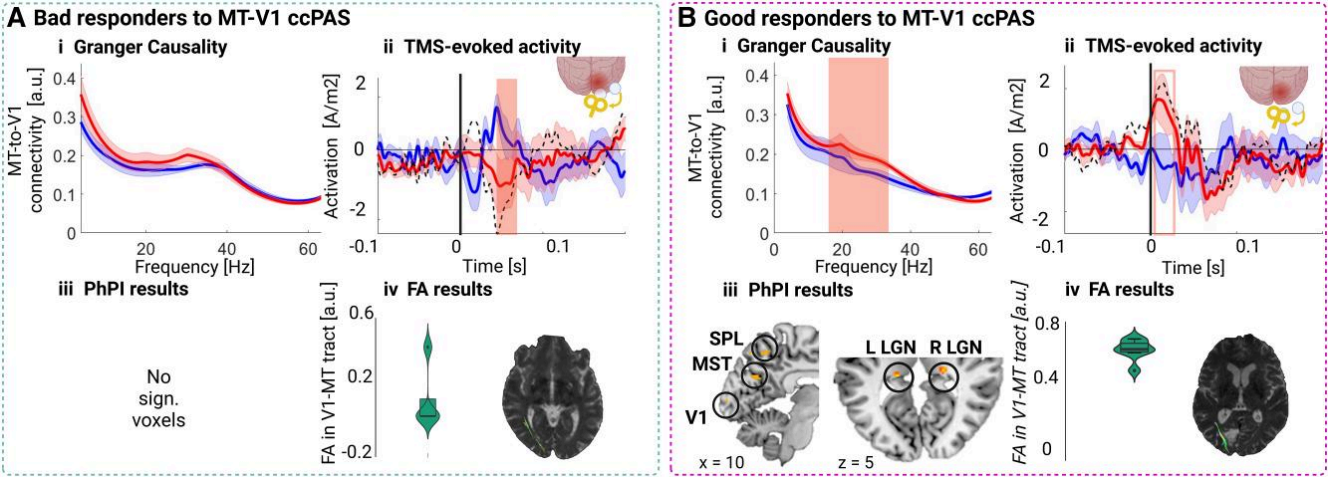
**Subgroup results**. [**A**(**i**–**iv**)] Electrophysiological and neuroimaging characterization of bad responders. (**i**) Spectral Granger Causality of the re-entrant MT-to-V1 inputs after single pulse transcranial magnetic stimulation (TMS) on V1. (**ii**) Ipsilesional V1 local source activity (LSA) after single pulse TMS over V1 (pulse at t = 0). (**iii**) Physio-Physiological Interaction (PhPi) results highlighting brain regions that significantly interact with the MT-V1 functional connection; *P* < 0.05 with false discovery rate (cluster) corrections. (**iv**) Diffusion weighted imaging derived mean fractional anisotropy (FA) in the ipsilesional V1-MT tract and one representative patient. [**B**(**i–iv**)] Electrophysiological and neuroimaging characterization of good responders. (**i**) Spectral Granger Causality of the re-entrant MT-to-V1 inputs after single pulse TMS on V1. (**ii**) Ipsilesional V1 LSA after single pulse TMS over V1 (pulse at t = 0). (**iii**) PhPI results. (**iv**) Diffusion weighted imaging derived mean FA in the ipsilesional V1-MT tract and one representative patient. The blue/red lines and shaded areas represent the mean and the standard error of the mean pre- and post-Backward ccPAS, respectively. Red vertically shaded areas indicate periods of significant differences between pre and post using frequency-wise non-parametric permutation tests (10 000 permutations, *P* < 0.05). ccPAS = cortico-cortical Paired Associative Stimulation; LGN = lateral geniculate nucleus; MT = middle temporal area; V1 = primary visual cortex.

First, the Granger Causality results were significantly enhanced in the High Beta frequency range (19–35 Hz) in good responders only (frequency-wise permutation test, 10 000 permutations, *P* < 0.05). Conversely, no significant changes were observed in the bad-responders group [Fig. 3A(i)]. Additionally, LSA over V1 in response to a TMS single pulse was significantly inhibited in an early time-window (between 40 and 60 ms) in the bad-responders group, while a trend for an increase was observed in the good-responders group [Fig. 3B(ii)].

The results of the PhPI analysis showed that the V1-MT functional pathway was functionally disconnected to the rest of the brain in the bad responders to MT-V1 ccPAS [Fig. 3A(iii)]. In contrast, the pathway of interest was significantly co-varying with the activity in the bilateral LGN, in the ipsilateral medial superior temporal (MST) area and in the ipsilesional superior parietal lobule (SPL), in the good responders [Fig. 3B(iii) and Supplementary Table 1]. Finally, since this study examines patients with acquired brain lesions, in whom parts of the tracts connecting V1 to MT might have been damaged by the ictal event, we computed the mean FA in the MT-V1 tracts in the lesioned hemisphere to index structural integrity of the V1-MT pathway. The results revealed significantly higher FA in good responders compared with bad responders [*t*(6) = 6.16. *P* < 0.001; Fig. 3A(iv) and B(iv)]. Note the lesion volumes and time since stroke did not differ between the two groups [lesion size: *t*(13) = −0–72, *P* = 0.49, time since stroke: *t*(13) = −1.65, *P* = 0.12, independent *t*-tests].

### Predictors of MT-V1 ccPAS effects

To strengthen the results from the median split and to understand the weights of each of these variables in the inter-individual variability of the response to MT-V1 ccPAS, we conducted a stepwise regression model with the following predictors which appeared to distinguish bad from good responders: the changes in Granger Causality of the re-entrant MT-to-V1 connection, the changes in local perilesional source activity in response to TMS and the beta-weights extracted from the PhPI analyses in the ipsilesional LGN. Additionally, we added the individual V1-MT FA values into the model.

The stepwise regression model was significant [*F*(2,13) = 10.23, *P* = 0.004] and explained a relevant amount of the variance (R^2^ = 0.60). The MT-to-V1 Granger Causality and the FA were retained as significant predictors [Granger Causality: *t*(13) = −2.8, *P* = 0.019, FA: *t*(13) = −3.0, *P* = 0.013]. Importantly, the two predictors were not correlated with each other (r = 0.15, *P* = 0.61).

**Figure 4:**
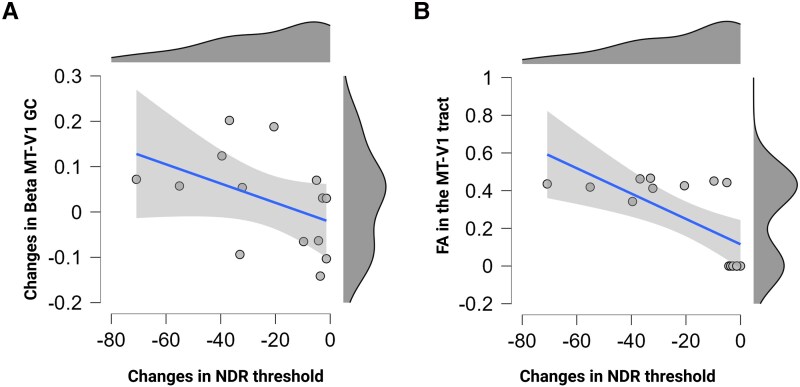
**Stepwise regression**. (**A** and **B**) Correlation plots and distribution plots of the relationship between the changes in normalized direction range (NDR) thresholds and the changes in MT-V1 Granger Causality (GC) in beta (**A**) or in fractional anisotropy (FA) in the V1-MT tract (**B**). MT = middle temporal area; V1 = primary visual cortex.

For illustrative purposes, Fig. 4A and B shows the correlation plots between the changes in NDR thresholds and the changes in MT-V1 Granger Causality in the beta band and between the changes in NDR thresholds and in FA values in the V1-MT tract, respectively.

## Discussion

**Figure 5:**
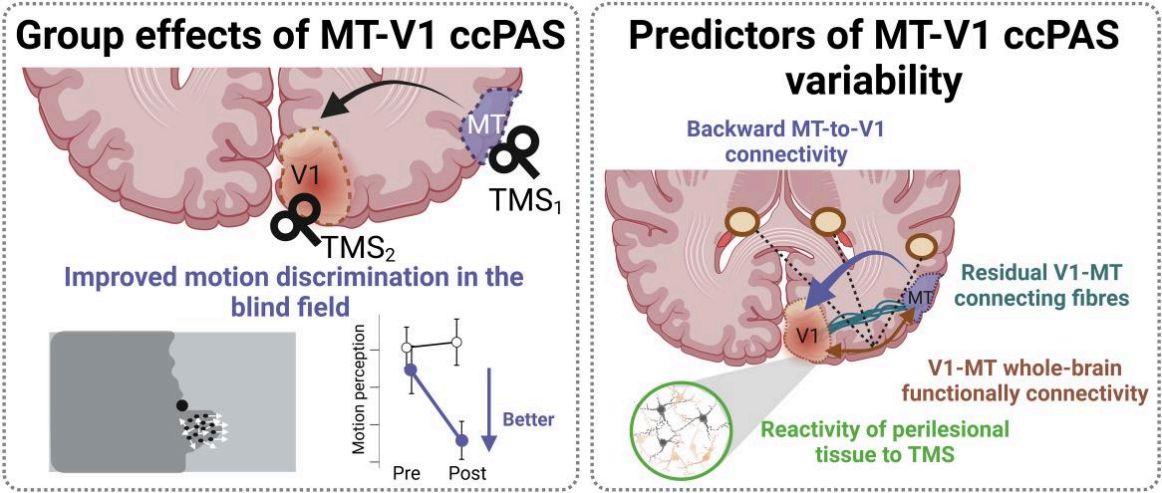
**Summary of the present findings: the induced ccPAS effects and its individual predictors**. ccPAS = cortico-cortical Paired Associative Stimulation; MT = middle temporal area; V1 = primary visual cortex.

In the present study, we used a combined MRI, TMS-EEG and behavioural paradigm to investigate whether the residual re-entrant MT-to-V1 pathway in patients with lesioned visual cortex can be modulated to enhance motion discrimination at the border of their visual field. Additionally, we aimed to test whether residual structural connectivity or functional reactivity of the task-relevant pathway can accurately predict how much a single patient will benefit from the ccPAS intervention. A significant group-level improvement in motion discrimination in the blind visual field was only reported after MT-V1 ccPAS. The individual improvements were found to be predicted by the level of structural integrity along the V1-MT pathway (see Fig. 5 for a graphical outline of the present results).

Evidence from macaques and humans has shown that back-projections from extrastriate areas to V1 are crucial for motion discrimination.^52,53,65^ In an earlier study, Romei *et al*.,^42^ applied MT-V1 ccPAS in healthy subjects and found improved motion coherence discrimination, suggesting plastic changes within this pathway. We provided additional support for this finding, and revealed for the first time, an associated increase in top-down MT-to-V1 connectivity only after MT-V1 ccPAS in patients with V1 damage, in proportion with the remaining structural fibres. Our results at the group level suggest that despite V1 damage, Hebbian rules of plasticity appear to apply in patients, with significant improvements in motion discrimination. Beyond our current and past (on a healthy cohort^43^) findings on ccPAS in visual motion discrimination, this technique has demonstrated its efficacy on healthy individuals in other domains. For instance, Quartarone *et al*., showcased the utility of ccPAS in enhancing motor performance in healthy individuals, suggesting potential applications in motor rehabilitation.^66^ Furthermore, Pellicciari *et al*., explored the application of ccPAS to modulate working memory performance in healthy individuals.^67^ Such research underscores the broad utility of ccPAS in diverse neurological applications, ranging from motor rehabilitation to cognitive therapy. Interestingly enough, we did not report any evidence for bidirectional long-term potentiation or depression of networks conversely to previous literature.^68,69^ One potential explanation might arise from our stimulation parameters which did not allow different postsynaptic neurons to be recruited, using a constant inter-pulse interval and pulse shape.

Notably, inter-individual variability in the response to MT-V1 ccPAS was high compared with healthy participants. To understand the bases of this variability, we compared spectral Granger Causality of the targeted pathway (i.e. the re-entrant MT-to-V1 connection) in the group of patients who had the largest improvements during the task after MT-V1 ccPAS to ones with reduced improvement (bad responders). While the bad responders did not show any significant change in the MT-to-V1 inputs, the good responders showed the expected increase in the re-entrant top-down MT-V1 connections in the beta band. This enhanced High Beta connectivity reflects synaptic plasticity along this pathway. The beta band’s role in cognitive and motor functions is well-documented, with increases in beta connectivity associated with feedback sensorimotor processing in particular.^70,71^ Our results suggest that similar mechanisms might be at play in the visual system, mediating top-down processing.^72^ As a matter of fact, the modulation of beta oscillations in the MT-to-V1 pathway is reported to be crucial for top-down control of motion perception. The study by Richter *et al*.^73^ revealed that top-down beta band influences from higher visual areas enhance bottom-up gamma-band processes in V1, suggesting a mechanism where beta oscillations modulate motion perception through cross-frequency interactions. Furthermore, beta oscillations in the early visual cortex convey behavioural context, affecting the processing of motion stimuli.^74^ The beta band’s role, particularly its alterations post-ccPAS, is a noteworthy finding given its association with cortical excitability and functional connectivity in motor^71^ and visual systems.^75^ The changes in beta Granger Causality following TMS, therefore, may reflect a state of heightened readiness in the visual cortex to engage in the processes of reorganization required for visual field recovery. These changes were only visible when looking at the EEG activity in response to TMS and not the EEG activity associated with the motion direction discrimination task. One can speculate that during the task, a homeostatic plasticity mechanism is triggered, cancelling the induced ccPAS effects.^76^ Interestingly, in our study on healthy subjects,^43^ changes were observed in the alpha frequency band. This shift towards the beta band in patients can be attributed to poststroke neural dynamics, where functional reorganization in cerebral networks leads to changes in oscillatory patterns. This reorganization often results in an altered excitatory-inhibitory balance within neural networks, influencing oscillatory activity in both alpha and beta bands.^77^ Beta oscillations in particular have been shown to be significantly relevant in poststroke functional recovery.^78,79^ Specifically, Thibaut *et al*.^79^ found that increased High-Beta power in the affected hemisphere correlates with improved motor performance, suggesting a compensatory mechanism due to the lesion-induced excitability imbalance. This shift from alpha to beta band in lesioned environments could signify the brain’s adaptive response to injury, emphasizing the role of beta oscillations as a potential biomarker in chronic stroke recovery.^77^

While the behavioural improvements induced by MT-V1 ccPAS were predicted by the increase in re-entrant MT-to-V1 beta inputs, they were also partially explained by the residual structural connectivity between the ipsilesional MT and V1 measured with DWI. FA within the MT-V1 tracts can be seen as a marker of structural integrity, which is essential for efficient signal transmission and is likely critical for the effectiveness of ccPAS.^80^ The link between microstructural features, in particular, tract myelination and cortico-cortical interactions has already been shown in multimodal studies.^81,82^ The presence of intact fibre tracts may facilitate the induction of plastic changes in response to associative stimulation protocols. The bad responders had lower FA values in the MT-V1 pathway, which was also functionally disconnected to the rest of the brain during motion discrimination as shown in the fMRI findings. Conversely, this pathway was coupled to other relevant visual areas in the good responders such as the LGN or MST. Altogether, these results provide strong evidence that MT-V1 ccPAS directly recruits the remaining MT-to-V1 synaptic connections in lesioned brains and that these two measures especially, residual FA and directed connectivity changes, could be used more generally as predictors of efficacy when an intervention targets a specific pathway.

Intriguingly, these two variables captured distinct dimensions of the injured brain’s response to ccPAS. Functional connectivity was enhanced in some patients despite their low FA, and reciprocally. Microstructural alterations do not necessarily go along with impaired functional connectivity and reciprocally.^83,84^ This discrepancy is due to factors such as the compensatory reorganization of neural networks or differences in the integrity of other indirect white matter tracts between the areas.^85^ These results align with the literature emphasizing the complex structure-function interplay in the context of neural repair and rehabilitation,^86^ suggesting that structural integrity (measured with residual FA) would not be enough to satisfyingly predict the outcome of MT-V1 ccPAS. The dynamic nature of functional connectivity in response to interventions highlights the relevance of considering both structural and functional aspects in a complementary fashion to optimally design rehabilitation protocols (see the summary of our results in Fig. 5). Extrapolating these results to any other pathway-specific plasticity inducing interventions, we argue that residual FA and functional connectivity changes could be efficient predictors of intervention outcomes and more generally of stroke recovery.

In sum, our study lends substantial support to the adoption of a multimodal index that synergistically exploits both structural and functional metrics to enhance the predictive accuracy of rehabilitation outcomes following Backward ccPAS intervention in stroke patients. This innovative approach is directly related to the field of precision medicine,^87^ which aims to tailor treatments to each individual’s characteristics, in particular, in the field of poststroke visual recovery.^88,89^ By establishing a precise neural and behavioural profile of each individual, it might be possible to accurately predict treatment efficacy.^90^ Complementarily, parts of the variability we measured in response to MT-V1 ccPAS might not only come from the residual white matter tracts or from the differences in effective connectivity, but also from the trial-by-trial difference in brain state surrounding ccPAS paired pulses. One parameter that could be improved without disturbing timings for STDP induction is the rigid repetition rate of the pairs of TMS pulses. Inspired by the pulsed alpha inhibition theory,^91^ MT-to-V1 ccPAS effects could be strengthened by triggering each paired pulse stimulation based on the optimal phase of the on-going EEG derived alpha activity.

Some limitations associated with this study should be considered. First, although similar to the sample sizes found in comparable studies,^44,92,93^ the limited sample size in this study prevents the results from being generalized to all cortically blind stroke patients. Second, our sample of patients were relatively heterogenous in terms of severity, age and recovery stage. In conclusion, this study provides encouraging results, showing that STDP can be triggered in lesioned visual systems and can result in behaviourally relevant effects, in proportion to enhanced functional connectivity and residual structural integrity of the pathway. To validate the prognostic values of these results, future research must longitudinally assess these multimodal indices in various cohorts and different stages of stroke recovery. The possibility to apply such markers for state-dependent ccPAS in clinical settings on a larger and heterogenous cohort will also necessitate thorough examination.^94^

## Supplementary Material

awaf043_Supplementary_Data

## Data Availability

Raw data were generated at the Sion and Geneva facilities, as reported in the ‘Materials and methods’ section. Derived data supporting the findings of this study are available from the corresponding author on request.
