# Supplementary material for: Pathway-dependent brain stimulation responses indicate motion processing integrity after stroke
Source: Brain. 2025 Feb 4;148(7):2361–72. doi: 10.1093/brain/awaf043 (PMC12233552; doi:10.1093/brain/awaf043)
Supplement: awaf043_Supplementary_Data [file awaf043_supplementary_data.pdf]

Supplementary Materials

Functional Localizer

To precisely target individual V1 and MT areas, we used a standard fMRI MT localizer task performed prior the TMS-EEG session (Figure 1A). During the functional localizer, the screen displayed radially moving dots alternating with stationary dots (see e.g. Sack et al., 2007). A block design alternated six 15 s blocks of radial motion with six blocks featuring stationary white dots in a circular region on a black background. This region subtended 25° visual angle, with 0.5 dots per square degree. Each dot was 0.36° diameter. In the motion condition the dots repeatedly moved radially inward for 2.5 s and outward for 2.5 s, with 100% coherence, at 20°/s measured at 15° from the center. Participants were passively looking at the screen and were asked to focus on a fixation point located in the middle of the screen. The resulting activation map and the individual T1-weighted image were entered into the neuro-navigation software to define the coil positions (Figure 1A, bottom left panel). The mean coil positions for V1 were -16±9, -86±8, -5±22 and for MT were 66±9, -55±9, -7±17 (coordinates x, y z, MNI space). The coil was held tangentially to the scalp with the handle pointing upwards and laterally at a 45° angle to the sagittal plane.

Granger Causality's computation

Granger causality is a measure of linear dependence, which tests whether the variance of error for a linear auto-regressive (AR) model estimation of a signal *x(t)* can be reduced when adding a linear model estimation of a second signal *y(t)*. If this is true, signal *y(t)* has a Granger causal effect on the first signal *x(t)*, i.e., independent information of the past of *y(t)* improves the prediction of *x(t)* above and beyond the information contained in the past of *x(t)* alone. The term independent is emphasized because it creates interesting properties for Granger Causality, such as that it is invariant under rescaling of the signals, as well as the addition of a multiple of *x(t)* to *y(t)*. The measure of Granger Causality is non-negative, and zero when there is no Granger causality. According to the original formulation of Granger Causality, the measure of Granger Causality from *y(t)* to *x(t)* is defined as:

$$F_{y\to x} = \ln\left(\frac{\mathrm{Var}(e_1)}{\mathrm{Var}(e_2)}\right)$$

Which is 0 for Var(e_1) = Var(e_2) and a non-negative value for Var(e_1) > Var(e_2). Note that Var(e_1) ≥ Var(e_2) always holds, as the model can only improve when adding new information. Under fairly general conditions, $F_{y\to x}$ can be decomposed by frequency if the two AR models in time domain are specified as:

$$\mathrm{x(t)} = \sum_{k=1}^{p}\left[A_{k_{xx}}x(t-k) + A_{k_{xy}}y(t-k)\right] + \sigma_{xy}$$

$$\mathrm{y(t)} = \sum_{k=1}^{p}\left[A_{k_{yy}}y(t-k) + A_{k_{yx}}y(t-k)\right] + \sigma_{yx}$$

In each equation the reduced model can be defined when each signal is an AR model of only its own past, with error terms σ_{xx} and σ_{yy} . We can then define the variance-covariance matrix of the whole system as:

$$\begin{bmatrix} \Sigma_{xx} & \Sigma_{xy} \\ \Sigma_{yx} & \Sigma_{yy} \end{bmatrix}$$

Where $\Sigma_{xx} = Var(\sigma_{xx})$, etc. Applying a Fourier transform to these equations, they can be expressed as:

$$\begin{pmatrix} A_{xx}(\omega) & A_{xy}(\omega) \\ A_{yx}(\omega) & A_{yy}(\omega) \end{pmatrix} \begin{pmatrix} x(\omega) \\ y(\omega) \end{pmatrix} = \begin{pmatrix} \varepsilon_1(\omega) \\ \varepsilon_2(\omega) \end{pmatrix}$$

Rewriting this as:

$$\begin{pmatrix} x(\omega) \\ y(\omega) \end{pmatrix} = \begin{pmatrix} H_{xx}(\omega) & H_{xy}(\omega) \\ H_{yx}(\omega) & H_{yy}(\omega) \end{pmatrix} \begin{pmatrix} \varepsilon_1(\omega) \\ \varepsilon_2(\omega) \end{pmatrix}$$

Where $\mathrm{H}(\omega)$ is the transfer matrix, the spectral matrix is then defined as:

$$S(\omega) = H(\omega) \sum H^*(\omega)$$

Finally, assuming independence of the signals x and y, and $\sum_{xy} = \sum_{yx} = 0$, we can define the spectral Granger Causality as:

$$F_{y\rightarrow x}(\omega) = \ln\left(\frac{S_{xx}(\omega)}{H_{xx}(\omega)\sum_{xx} H^*_{xx}(\omega)}\right)$$

Granger Causality was then computed between the four clusters (V1, MT, IPS and FEF), in a frequency range of 1-45 Hz and with a definition of 3 Hz, in order to explore the role of different rhythms in the causal connectivity between brain regions. Note that the use of Granger causality on source-projected EEG data reduces the problem of signal mixing and volume conduction, probably because Granger causality reflects causal, i.e. time-delayed, interactions, and explicitly discards instantaneous interactions resulting from signal mixing [2–4].

Task-EEG preprocessing and analyses:

All the preprocessing steps have been run on MATLAB, using the EEGLAB toolbox [5]. Data was re-referenced to the average of all channels, band-passed filtered between 1 Hz and 80 Hz, notch filtered between 48 and 52 Hz, and divided in epochs of 1.5 s length. Visual inspection was used to remove explicit artifacts among channels and trials, followed by the reconstruction of dropped channels and epochs. Ultimately, an Independent Component Analysis was applied to the down-sampled data (1000 Hz) to remove electrophysiological interferences, such as eyeblinks or muscle artifacts. Brainstorm software [6] together with OpenMEEG BEM plugins were used to perform source level reconstruction of EEG data. First, the cortex and head mesh (15,000 and 10,000 vertices respectively) of the patient were generated using the automated MRI segmentation routine of FreeSurfer [7]. The forward model was then computed using the symmetric Boundary Element Method developed in the open OpenMEEG freeware, using default values for conductivity and layer thickness [8]. The source level activation was computed using a Minimum norm imaging linear method with sLORETA as inverse model. The dipole orientation of the source model has been defined as constrained to the cortex surface. The covariance matrix was computed from the concatenated epoch baselines, e. g. the recorded activity before the onset of each trial [-0.5 -0.005] s. The source points belonging to specific areas of interest (i.e., V1 and MT), were individually defined according to the fMRI localizer recordings performed before the EEG acquisitions. We first examined the time-frequency content of the V1-MT pathway during the CDDI task at the source level. All metrics involving a frequency domain decomposition were calculated through Morlet wavelets between 1 and 60 Hz. Cluster-based permutation tests were performed in each frequency bin for alpha (8-12Hz), beta (15-25Hz), low gamma (35-45Hz) and high gamma (51-60Hz) on two time-windows [0.001-400ms] and [401-800ms]. Additionally, we computed spectrally resolved Granger causality 68 using the individual sources encompassing the ipsilesional V1 and MT as described for the TMS-EEG data in the supplementary materials ("Granger Causality's computation").

fMRI analyses

The pre-processing steps included correction for field inhomogeneity, slice timing correction, motion correction and unwarping. Then, the structural image of each patient was co-registered to the mean realigned EPI volume. The co-registered T1-weighted image was then normalized to the Montreal Neurological Institute (MNI) reference space using the unified segmentation approach [9]. The resulting deformation parameters were applied to the individual EPI volumes which were then smoothed using an isotropic 4 mm full-width half-maximum (FWHM) Gaussian kernel.

For all datasets, we modelled a GLM using two regressors based on the patients' trial by trial accuracy in line with our staircase procedure (correct (74.63 (+/- 9.6) trials/incorrect (25.99 (+/- 9.7) trials)). Regressors were modelled as series of events (representing individual epochs) convolved with a canonical hemodynamic reference waveform. Low-frequency confounds were controlled by high-pass filtering at 1/128 Hz and head-movement estimates derived from the realignment procedure served as additional covariates of non-interest. Voxel-wise parameter estimates for all conditions and each covariate resulting from the least mean squares fit of the model to the data were computed. For the group analysis, left-side lesions were mirrored to the right hemisphere.

Analyses of the Diffusion Weighted Imaging data

A denoising step was firstly applied via the dwidenoise function (MRtrix), followed by correction of Gibbs ringing artefact via mrdegibbs (MRtrix) [10]. Images were then corrected for motion, susceptibility induced fields, eddy-current induced distortions, and bias field via the FSL functions topup [11], eddy_openmp [12,13] and fast [14]. Probability maps for CSF, grey and white matter were estimated from the T1-weighted image via the fast function (FSL) and then registered to the average b0 image using ANTs (Avants et al., 2014). Fibre orientation distribution function was derived at the voxel level from multi-shell multi-tissue constrained spherical deconvolution and then used to compute whole-brain probabilistic tractography via second-order integration over fibre orientation distribution (iFOD2) [16]. The algorithm stopped once 10 million streamlines were generated. Each streamline was then weighted based on spherical-deconvolution informed filtering of tractograms (SIFT2, MRtrix) [17].

Supplementary Table 1

Regions	F values	Z max	Cluster extent	MNI coordinates (x;y;z)
R Lateral Geniculate Nucleus	13.4	3.35	31	4;-76;-3
L Lateral Geniculate Nucleus	12.7	3.26	19	3;46;3
R Superior Parietal Lobe	11.7	3.39	17	-29;46;28
R Medial Superior Temporal				
R Primary Visual Cortex				

Supplementary Table S1: *Significant clusters in the PhPI analyses with the V1-MT pathway as seed*

Supplementary Figure S1: Baseline stability of motion discrimination performance

At the group level, no difference was observed between the performances measured on the day before and on the actual first day of experiment ($t_{(15)}$ = -1.54, p = 0.14), ensuring stable performance at baseline.

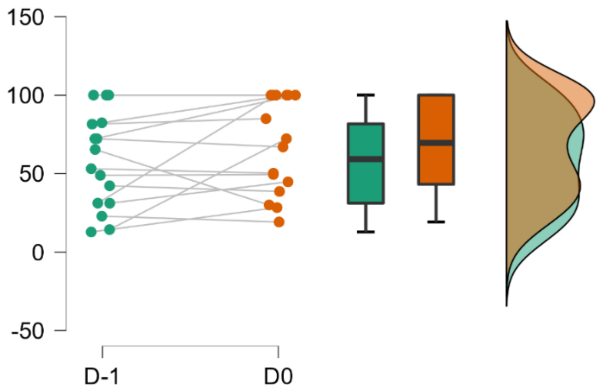

Supplementary Figure S1: *A paired t-test revealed no significant difference between NDR thresholds measured the day before the first session of the experiment and the first "Pre" ccPAS measurement (t(15) = -1.54, p = 0.14).*

Supplementary Figure S2: Motion awareness and reaction time

For exploratory purposes, we inspected potential changes in motion awareness and in reaction times. Supplementary Figure 2A shows the group distribution of the Pre/Post difference in motion awareness for the trials correct trials only, for the two ccPAS conditions. The rmANOVA did not show any significant main effects or interaction (Time effect: $F(1,8) = 1.8$, $p = 0.21$; ccPAS effect: $F(1,8) = 0.07$, $p = 0.8$; Time by ccPAS interaction: $F(1,8) = 3.3$, $p = 0.1$). Supplementary Figure 2B reports the group distribution of the Pre/Post difference in response times for the correct trials of the two ccPAS conditions. While we observe a general Time effect ($F(1,8) = 12.7$, $p = 0.007$), no significant ccPAS type by Time interaction was found ($F(1,8) = 0.48$, $p = 0.51$).

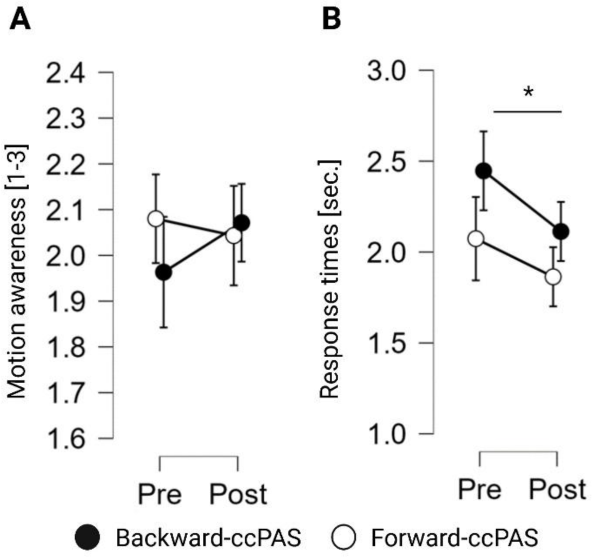

Supplementary Figure S2: A: *Distribution of the Pre/Post difference in motion awareness for the correct trials;* ***B****: Distribution of the Pre/Post difference in reaction time for the correct trials.*

Supplementary Figure S3: Changes in effective connectivity for Forward ccPAS

Granger Causality analysis at Forward ccPAS group level showed a task-irrelevant significant increase of direct V1-to-MT effective connectivity in the Alpha/Beta frequency range (7-21 Hz) after V1 single pulse stimulation, an a increase of direct MT-to-V1 effective connectivity in the High Beta frequency band (27-35 Hz) after MT single pulse stimulation. No significant changes were observed elsewhere.

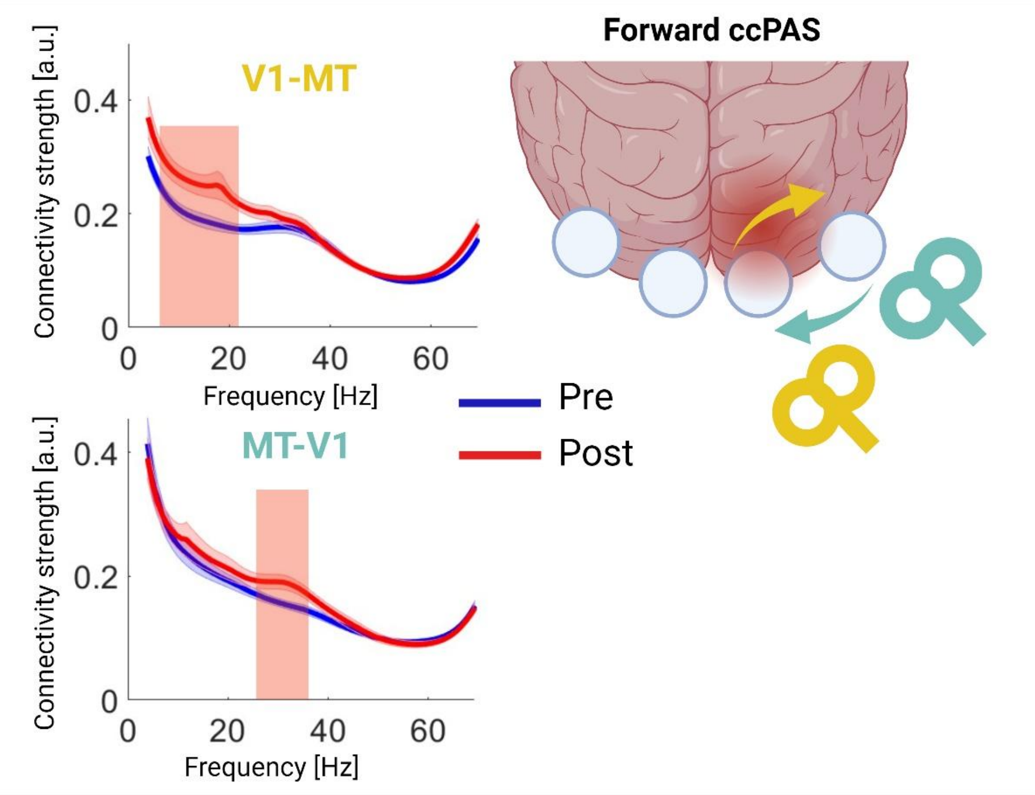

Supplementary Figure S3: *Forward ccPAS group-level Spectral Granger Causality of V1-MT network when TMS was given to V1 (in yellow) or to MT (in green). On the left, the significant frequency band for the 2 pathways that showed a significant change in any configuration: significant increase in the alfa/beta (7-21 Hz) band in V1-to-MT after V1 was stimulated with single pulse TMS, significant increase in high beta (27-35 Hz) in MT-to-V1after MT was stimulated with single pulse TMS. Shaded areas indicate periods of significant differences between PRE and POST using non-parametric, cluster-based, permutation tests (10000 permutations, $p<0.05$), excluding frequency-wise outliers (>90th percentile).*

Supplementary Figure S4: Heatmap visualization of lesions' frequency overlaid on an MNI template brain

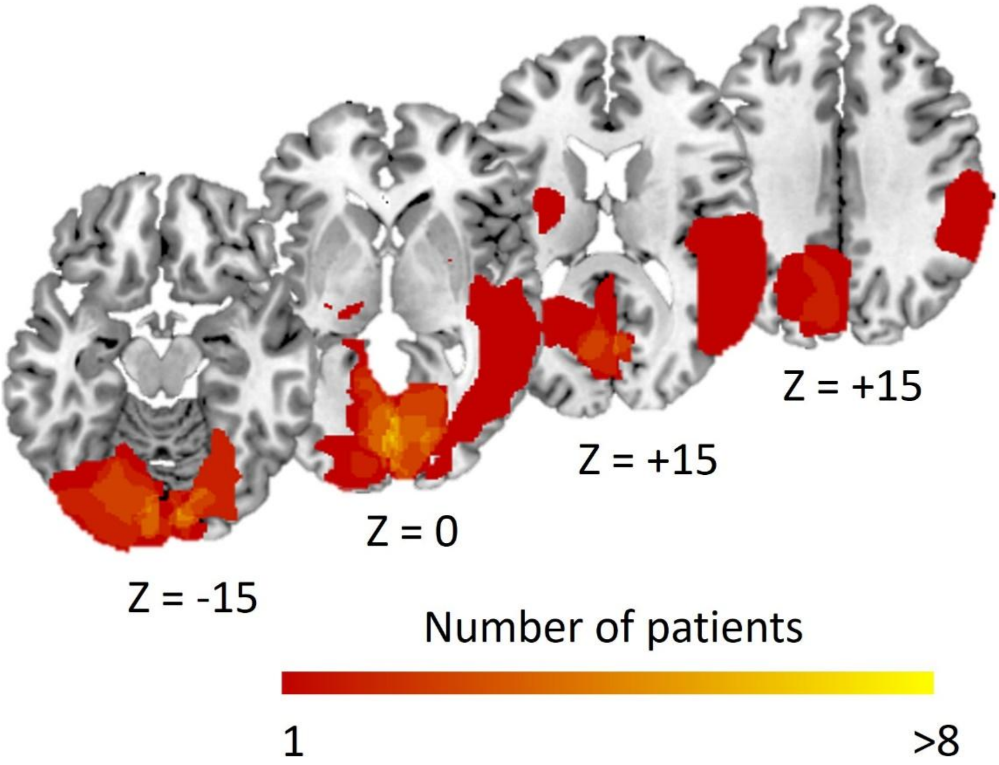

Figure S4: *Heatmap visualization of lesions' frequency overlaid on an MNI template brain.*

Supplementary Figure S5: Task-EEG results

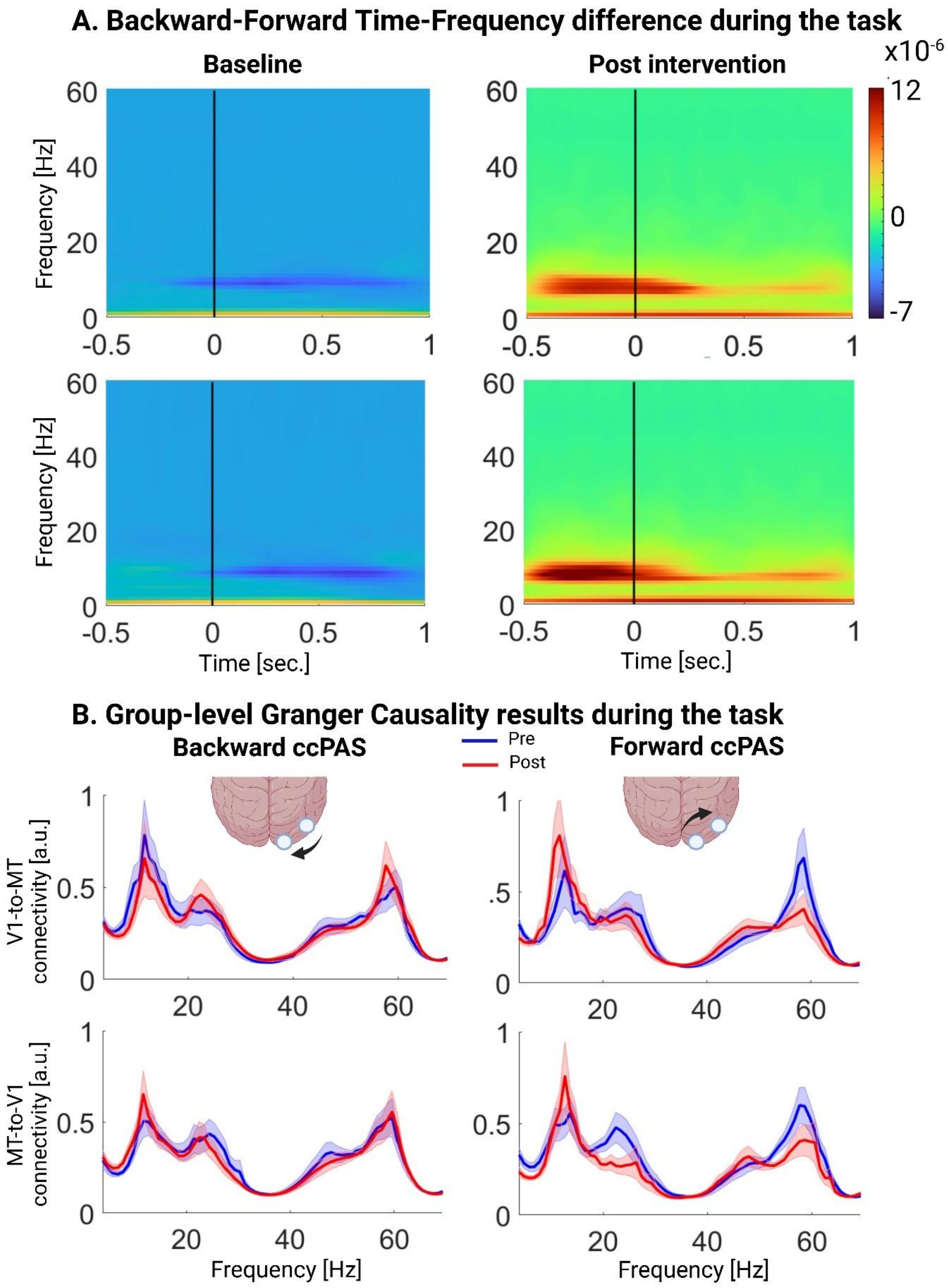

Figure S5A: *Backward minus Forward ccPAS Difference in Time-Frequency content at baseline (left) and after the intervention (right); B Group-level Spectral Granger Causality of V1-MT network during the motion direction discrimination task before (blue) and after (red) Backward ccPAS (left) and Forward-ccPAS (right). None of the comparisons were significant (cluster-based permutation tests).*

Bibliography

1. Sack AT, Kohler A, Bestmann S, et al. Imaging the brain activity changes underlying impaired visuospatial judgments: simultaneous FMRI, TMS, and behavioral studies. *Cereb Cortex N Y N 1991*. 2007;17(12):2841-2852. doi:10.1093/cercor/bhm013

2. Bastos AM, Vezoli J, Bosman CA, et al. Visual areas exert feedforward and feedback influences through distinct frequency channels. *Neuron*. 2015;85(2):390-401. doi:10.1016/j.neuron.2014.12.018

3. Michalareas G, Vezoli J, van Pelt S, Schoffelen JM, Kennedy H, Fries P. Alpha-beta and gamma rhythms subserve feedback and feedforward influences among human visual cortical areas. *Neuron*. 2016;89(2):384-397. doi:10.1016/j.neuron.2015.12.018

4. West TO. Measuring directed functional connectivity using non-parametric directionality analysis: Validation and comparison with non-parametric Granger Causality. Published online 2020:19.

5. Delorme A, Makeig S. EEGLAB: an open source toolbox for analysis of single-trial EEG dynamics including independent component analysis. *J Neurosci Methods*. 2004;134(1):9-21. doi:10.1016/j.jneumeth.2003.10.009

6. Tadel F, Baillet S, Mosher JC, Pantazis D, Leahy RM. Brainstorm: A User-Friendly Application for MEG/EEG Analysis. *Comput Intell Neurosci*. 2011;2011:1-13. doi:10.1155/2011/879716

7. Reuter M, Schmansky NJ, Rosas HD, Fischl B. Within-subject template estimation for unbiased longitudinal image analysis. *NeuroImage*. 2012;61(4):1402-1418. doi:10.1016/j.neuroimage.2012.02.084

8. Gramfort A, Papadopoulo T, Olivi E, Clerc M. OpenMEEG: opensource software for quasistatic bioelectromagnetics. *Biomed Eng OnLine*. 2010;9(1):45. doi:10.1186/1475-925X-9-45

9. Ashburner J, Friston KJ. Unified segmentation. *NeuroImage*. 2005;26(3):839-851. doi:10.1016/j.neuroimage.2005.02.018

10. Veraart J, Novikov DS, Christiaens D, Ades-Aron B, Sijbers J, Fieremans E. Denoising of diffusion MRI using random matrix theory. *NeuroImage*. 2016;142:394-406. doi:10.1016/j.neuroimage.2016.08.016

11. Andersson JLR, Skare S, Ashburner J. How to correct susceptibility distortions in spin-echo echo-planar images: application to diffusion tensor imaging. *NeuroImage*. 2003;20(2):870-888. doi:10.1016/S1053-8119(03)00336-7

12. Andersson JLR, Sotiropoulos SN. An integrated approach to correction for off-resonance effects and subject movement in diffusion MR imaging. *NeuroImage*. 2016;125:1063-1078. doi:10.1016/j.neuroimage.2015.10.019

13. Smith SM, Jenkinson M, Woolrich MW, et al. Advances in functional and structural MR image analysis and implementation as FSL. *NeuroImage*. 2004;23 Suppl 1:S208-219. doi:10.1016/j.neuroimage.2004.07.051

14. Zhang Y, Brady M, Smith S. Segmentation of brain MR images through a hidden Markov random field model and the expectation-maximization algorithm. *IEEE Trans Med Imaging*. 2001;20(1):45-57. doi:10.1109/42.906424

15. Avants BB, Tustison N, Johnson H. Advanced Normalization Tools (ANTS).

16. Tournier JD. Diffusion MRI in the brain – Theory and concepts. *Prog Nucl Magn Reson Spectrosc*. 2019;112-113:1-16. doi:10.1016/j.pnmrs.2019.03.001

17. Smith RE, Tournier JD, Calamante F, Connelly A. SIFT2: Enabling dense quantitative assessment of brain white matter connectivity using streamlines tractography. *NeuroImage*. 2015;119:338-351. doi:10.1016/j.neuroimage.2015.06.092
